# Corneal Nodules and Possible Pathologies: A Case Series

**DOI:** 10.7759/cureus.20822

**Published:** 2021-12-30

**Authors:** Huey Chuin Kuan, En Yoo Ivan Cheng, Meng Hsien Yong, Wan Haslina Wan Abdul Halim, Othmaliza Othman

**Affiliations:** 1 Ophthalmology, Faculty of Medicine, Hospital Universiti Kebangsaan Malaysia, Kuala Lumpur, MYS; 2 Ophthalmology, Hospital Shah Alam, Shah Alam, MYS

**Keywords:** corneal nodule, corneal pathology, case series, herpetic stromal keratitis, phlyctenular keratoconjunctivitis, salzmann nodular degeneration, anterior segment optical coherence tomography

## Abstract

Corneal nodular lesions are not uncommon in clinical practice. Diagnosing and managing this condition can be challenging due to its variable causes. This article highlights three cases of corneal nodular lesions. A common clinical pathway for the diagnosis and treatment of cornea nodular lesions is discussed. Two young females and an elderly man presented with a unilateral corneal nodule of variable duration, which was further demonstrated on anterior segment optical coherence tomography (AS-OCT). Several diagnoses were made after thorough history and examination which include herpetic stromal keratitis, phlyctenular keratoconjunctivitis secondary to blepharitis, and Salzmann nodular degeneration. All cases were initiated on topical antibiotics and topical steroids with additional medication or surgical procedure onboard according to their clinical condition. The corneal nodules resolved with scarring after a period of treatment. In conclusion, corneal nodular lesions can be associated with various pathologies. Thorough history, examination, and appropriate investigations are needed to reveal the underlying causes. Serial anterior segment images and AS-OCT are useful to monitor progression and treatment response. Prompt diagnosis and initiation of treatment are crucial to prevent further complications.

## Introduction

Corneal nodular lesions are not uncommon in clinical practice. As the pathogenesis of corneal nodule formation varies with the underlying cause, the management differs based on the pathologies. To date, there is no report comparing corneal nodular lesions and their possible underlying etiologies. Here, we report three cases of nodular lesions on the cornea with different underlying pathologies. We also discuss a general approach in dealing with such lesions.

## Case presentation

Case 1

**Figure 1:**
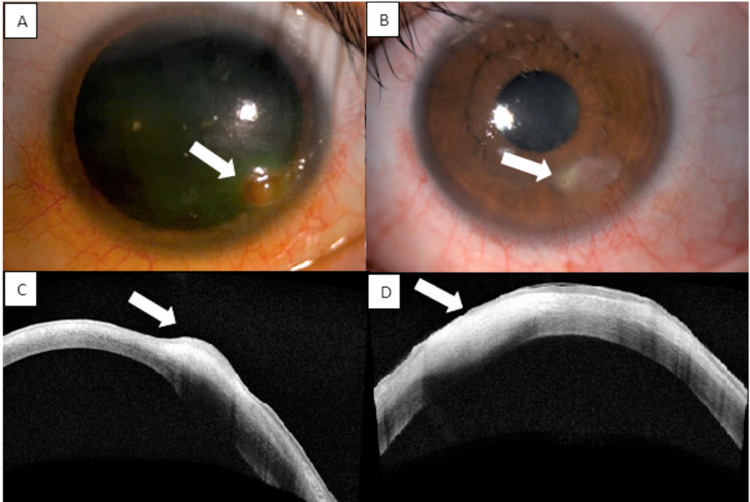
(A) Anterior segment image showing corneal nodular lesion on presentation and (B) after treatment (arrow). (C) AS-OCT image demonstrating the lesion confined to the anterior stroma. (D) Scarring after three months of treatment (arrow). AS-OCT: anterior segment optical coherence tomography

A 31-year-old female with an underlying treated cerebellopontine angle tumor complicated with left fifth and seventh cranial nerve palsies presented with left eye painless corneal opacity of one-week duration. Examination of the left eye revealed lagophthalmos and an absence of corneal sensation. There was diffuse superficial punctate keratitis over the inferior region and a nodular lesion at the inferotemporal area of the cornea, sparing the limbus. The nodule had a deep reddish hue, associated with overlying epithelial defect and superficial corneal vascularization (Figure 1A). The surrounding conjunctiva was mildly injected. At presentation, pinhole-aided visual acuity was 6/9 bilaterally. Anterior segment optical coherence tomography (AS-OCT) revealed a hyperreflective nodular lesion confined to the anterior stromal area (Figure 1C). Examination of the right eye was unremarkable.

She was started on topical levofloxacin every two hours and fixed-dose combination ointment dexamethasone-neomycin-polymyxin b at night for her left corneal lesion to treat both the infection and inflammation. However, the epithelial defect and vascularization worsened after treatment. Because of the reduced corneal sensation with worsening corneal neovascularization and new epithelial defect after initiation of steroid, oral acyclovir 400 mg five times per day was started for the treatment of herpetic stromal keratitis, and topical steroid was re-initiated after a week of oral acyclovir. Both oral acyclovir and topical steroid were tapered over three months. The lesion resolved with a scar and residual epithelial defect. However, at the end of three months, there was a recurrence of the elevated lesion over the scarred region with new deep vascularization and epithelial defect after topical steroid was tapered off. She was treated as a relapse of herpetic keratitis and was started on oral acyclovir 400 mg five times per day for two weeks and tapered over another three months, together with a topical steroid which was replaced by topical cyclosporin 0.5%. The left corneal lesion resolved with a well-defined scar and inactive vascularization (Figures 1B, 1D).

Case 2

An 11-year-old girl presented with left eye redness and an enlarging whitish lesion at the cornea for a month. She denied any eye pain, blurring of vision, or preceding ocular trauma. She had intermittent left eye itchiness and redness for the past five months which was partially relieved by topical antibiotics.

**Figure 2:**
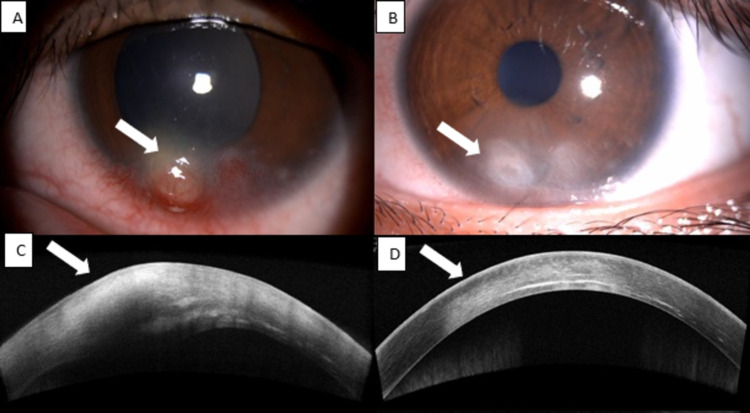
(A) Anterior segment image on presentation revealing the presence of a subepithelial nodule (arrow) at 6 to 7 o’clock of the left cornea, sparing but approaching limbus with surrounding subepithelial opacity, as evidenced by AS-OCT (C). (B, D) The corneal nodule resolved and scarred (arrow) after treatment. AS-OCT: anterior segment optical coherence tomography

On examination, visual acuity in both eyes was 6/12. There was a subepithelial nodule at 6 to 7 o’clock of the left cornea, sparing but approaching limbus (Figure 2A). The nodule was surrounded by subepithelial opacity with superficial and deep vascularization. There was no epithelial defect or infiltrate. Conjunctiva was injected with the presence of blepharitis and meibomianitis bilaterally. AS-OCT revealed a corneal nodule at the anterior stroma with heterogenous hyperreflectivity (Figure 2C). Corneal scraping for gram stain and culture revealed no growth. Mantoux test was negative. Blood investigations including a full blood count and C-reactive protein were normal. She was treated as left eye phlyctenular keratoconjunctivitis secondary to blepharitis and started on intensive topical levofloxacin, topical steroid, ointment fusidic acid, and oral doxycycline. The topical medications were tapered after two weeks of treatment. The nodule resolved and scarred after six weeks of treatment (Figures 2B, 2D).

Case 3

**Figure 3:**
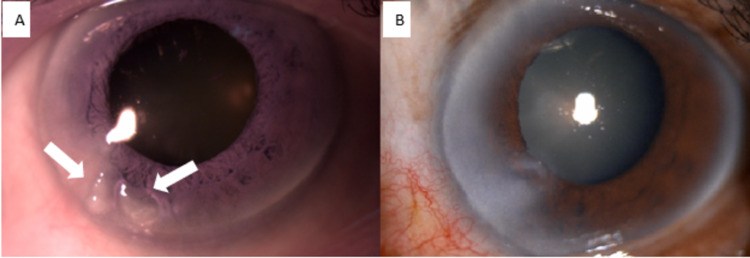
(A) Anterior segment image showing the presence of two discrete corneal nodules (arrow) at 7 o’clock without limbus involvement. (B) Corneal scarring after superficial keratectomy.

A 76-year-old male with no known medical illness was planned for a left eye cataract surgery. Examination of the left eye revealed nasal pterygium extending 2 mm from the limbus to the cornea. There were two corneal nodules. One nodule appeared to be coalescent of two smaller nodules seen at 7 o’clock, extending till the anterior stroma with no limbal involvement (Figure 3A). Dellen was found between the nodules. Otherwise, there was no epithelial defect, infiltration, or overlying vascularization. His aided visual acuity in both eyes was 6/9. On further investigation, he reported sustaining a blunt injury by a branch over his left eye at the age of 20 which he claimed did not affect his vision. A diagnosis of Salzmann nodular degeneration was made. He underwent pterygium excision and superficial keratectomy (Figure 3B). There was no recurrence of the nodule and he was scheduled for cataract surgery later.

## Discussion

Corneal nodules: The differential diagnoses

Different clinical conditions need to be considered when patients present with corneal nodules. Here, we consider a few differential diagnoses including infectious causes such as herpes simplex keratitis and non-infectious causes. Non-infectious etiology can be further subdivided into an inflammatory origin, for example, phlyctenular keratoconjunctivitis, marginal keratitis, and pyogenic granuloma, and non-inflammatory origin, for example, Salzmann nodular degeneration and spheroidal degeneration of the cornea.

Case 1: Herpetic Keratitis with Intracorneal Hemorrhage

Our patient presented with a corneal nodular lesion with the clinical diagnosis of herpetic stromal keratitis, as evidenced by reduced corneal sensation. It contrasts with the typical description of herpetic stromal keratitis which is associated with an opaque or white corneal lesion due to stromal infiltration, along with the presence of ulceration in necrotizing form and disc-shaped ground-glass due to stromal edema in disciform type [1]. Factors such as systemic disease, duration of symptoms, and previous treatment are believed to alter the appearance of the lesion during slit-lamp examination [2]. To date, there are no reports on the presence of corneal nodular lesions in herpetic keratitis.

Because of the reddish hue of the corneal nodule, intracorneal hemorrhage was highly suspected in this case. Corneal neovascularization is often seen in herpetic eye infections and therefore bleeding can occur from the corneal vascularization [3]. This was supported by AS-OCT which revealed that the lesion occurred at the sub-Bowman membrane area. In this case, hemorrhage extended anteriorly, mimicking a nodule. Another possible contribution to the corneal neovascularization in this patient could be the defective tear film lubrication over the ocular surface secondary to lagophthalmos. Pannus formation and neovascularization are known complications of severe dry eye [4]. In the presence of corneal neovascularization, stromal hemorrhage should be considered when managing corneal nodules.

Case 2: Phlyctenular Keratoconjunctivitis

Phlyctenular keratoconjunctivitis is a non-infectious cause of corneal nodular lesions. It is a cell-mediated delayed hypersensitivity response within the cornea or conjunctiva in response to a specific antigen such as a protein from *Mycobacterium tuberculosis*, *Staphylococcus*, and worm infestation [5]. Typical clinical conditions revealed inflammatory small, circumscribed nodules with vascularization at the corneoscleral limbus [5].

Thygeson described the varying size of the nodules from visible points to several millimeters in diameter [6]. The nodules usually involved the limbal region, bulbar, or even palpebral conjunctiva, which subsequently progressed to the cornea. It rarely occurs as pure keratitis. The level of corneal involvement is usually between the epithelium and Bowman’s membrane [7]. In our case, the patient had a preceding history of eye redness which would represent an unnoticed occurrence of the conjunctival lesion. AS-OCT demonstrated a similar location of the corneal nodule which involved the subepithelial area.

Case 3: Salzmann Nodular Degeneration

The mechanism of corneal lesion formation in Salzmann nodular degeneration remains unknown. However, it is believed that the disruption of barrier occurs between the epithelial basement membrane and stroma due to several conditions such as ocular trauma, chronic corneal irritation from meibomian gland disease, ocular surface disease, and non-inflammatory conditions, which include postoperative corneal surgery, corneal epithelial basement membrane dystrophy, and hard contact lenses [8]. Fibroblasts and myofibroblasts are activated and migrated anteriorly through disrupted epithelium basement membrane followed by deposition of disorganized extracellular matrix components which lead to nodule formation [9].

Clinically, bluish-white to gray subepithelial nodules are confined to the anterior stroma at any part of the cornea, more commonly at the periphery [10]. The nodules may vary from solitary nodules to large and irregular prominences because of the fusion of smaller nodules. It is more common among Caucasian women in a bimodal distribution, occurring in the fifth or eighth and ninth decades of life with bilateral involvement in 80% of the cases [11]. Our patient fulfilled the demographic prevalence of an elderly man with unilateral eye involvement. He had two nodules with one appearing to be a coalescent of smaller nodules seen at the corneal periphery.

Our patient also had a coexisting nasal pterygium with corneal nodules. Salzmann nodular degeneration is not found as a concurrent ocular surface condition associated with pterygium even though both Salzmann nodular degeneration and pterygium involve Bowman’s layer disruption and activation of stromal fibroblast [9,11]. Histologically, pterygium is thought to originate from altered limbal stem cells which extend centripetally to involve the cornea. On the other hand, Salzmann nodular degeneration does not involve limbal stem cells due to hyaline degeneration of collagen with excessive secretion of basement membrane-like material [8,9,11]. In our patient, pterygium could have been a chronic irritant to the ocular surface, which is a predisposing factor to the development of corneal nodules.

Corneal Nodules: Other Causes

For patients with preceding ocular trauma or infection, the presence of a corneal nodule should raise a suspicion of pyogenic granuloma. It is an aberrant wound healing process that does not involve pyogenic formation or granulomatous inflammation. Although uncommon, pyogenic granuloma of the cornea has been reported in the literature [12,13]. It is described as a rapidly growing, red, vascularized raised lesion with a smooth surface and pedunculated base. Patients with pyogenic granuloma share common clinical findings of preceding epithelial defects with corneal vascularization and chronic ocular irritant [13].

Another condition associated with corneal nodule is spheroidal degeneration of the cornea. It is a non-inflammatory elastotic degenerative condition of the cornea with extracellular proteinaceous deposits. It has a higher prevalence in areas with extreme temperature and environmental conditions such as low humidity, high wind, the presence of sand, and high levels of ultraviolet exposure [14]. Clinically, amber-colored, translucent, spheroid-like granules are seen at the conjunctival epithelium or peripheral cornea in the superficial stroma, Bowman’s membrane, and subepithelium. The interpalpebral region is usually involved where granules later coalesce to become nodules and involve the central cornea [15].

Clinical approach to corneal nodule

As a general approach in managing a patient presenting with a corneal nodule, the patient’s age is important. History of the current complaint should include the onset of the lesion and its associated symptoms such as irritation, tearing, eye redness, and blurring of vision. Symptoms depend on the location of the nodule which may range from a mild condition, such as photophobia and excessive lacrimation, to more severe complaints, such as reduction in visual acuity. Preceding ocular injury, surgery, and ocular surface disease should be identified as they may provide clues to the underlying pathology. Salzmann nodular degeneration occurs because of previous orbital disease [8], corneal surgery, and ocular trauma while the ocular infection is a risk factor for corneal pyogenic granuloma.

During the ocular examination, identification of the lid condition helps delineate the possible causes of corneal nodules. Phlyctenular keratitis and marginal keratitis are associated with blepharitis or meibomianitis [5]. Chronic irritation from ocular surface diseases such as dry eye and trichiasis are predisposing factors to pyogenic granuloma and Salzmann nodular degeneration. Other important factors include cornea sensation, presence of corneal vascularization, ocular surface evaluation by fluorescein staining, quality of tear film, and location of the nodule with any limbal or conjunctiva involvement. Reduced cornea sensation is the hallmark for herpes simplex keratitis in which the sub-basal nerve plexus is diminished after herpes simplex infection [16].

Ocular investigations such as corneal scraping for gram staining and cultures should be performed when infection cannot be ruled out. Viral polymerase chain reaction (PCR) is more helpful in viral detection compared to cell culture. However, in cases with atypical presentation of viral herpes infection or those on previous or current antiviral treatment, the role of viral PCR might be limited [17]. Non-invasive imaging techniques such as AS-OCT aid in identifying the lesion, localizing the involvement of the corneal layer, and excluding any corneal thinning. It is also useful as an objective tool to monitor the treatment response. Serial anterior segment images also aid in evaluating the progression of nodules.

Systemic investigations are indicated when an infectious cause is suspected. Phlyctenular keratitis is highly associated with tuberculin protein, and investigations such as Mantoux test, tuberculosis quantiferon, or chest X-ray are required to rule out tuberculosis [5].

Treatment for corneal nodules should be tailored to their etiologies. Herpetic keratitis requires antiviral with concurrent administration of topical corticosteroid in stromal and endothelial subtypes. However, it does not cure the disease but shortens the symptom duration and maintains the virus in latency [1]. The risk of recurrence is reduced by 45% in patients with oral administration of acyclovir 400 mg during the first 12 months of treatment [18]. Topical corticosteroid administration reduces stromal inflammation progression and does not increase the risk of epithelial disease recurrence [19]. For marginal keratitis, topical corticosteroid helps control local inflammation. Blepharitis should be treated with local antibiotics, and lid hygiene should be emphasized. In severe non-responsive blepharitis, a systemic antibiotic such as doxycycline is warranted. Phlyctenular keratitis and pyogenic granuloma require topical antibiotics and steroids. It is important to treat concurrent eyelid inflammation as it may induce ocular surface inflammation [20]. Depending on the size of the lesion, the mainstay of treatment for pyogenic granuloma is surgical excision with subsequent treatment directed at removing the causative factors such as ocular surface disease and ocular irritants [12,13]. However, smaller lesions may respond to a topical steroid resulting in complete regression. In Salzmann’s nodular degeneration, ocular lubricant, warm compression, and a short course of topical steroids are sufficient for patients with mild symptoms [9]. Surgical intervention, primarily superficial keratectomy, may be required for those with symptomatic nodules obscuring visual axis or refractory cases [8,9].

## Conclusions

Corneal nodular lesions can be associated with various pathologies. A systematic approach with a thorough history, examination, and tailored investigation aids clinicians in providing appropriate treatment. Subsequent devastating complications including blinding sequelae need to be prevented.
